# Assessing the Relationship Between Digital Trail Making Test Performance and IT Task Performance: Empirical Study

**DOI:** 10.2196/49992

**Published:** 2024-06-14

**Authors:** Tanguy Depauw, Jared Boasen, Pierre-Majorique Léger, Sylvain Sénécal

**Affiliations:** 1 Tech3lab HEC Montréal Montréal, QC Canada; 2 Faculty of Health Sciences Hokkaido University Sapporo, Hokkaido Japan

**Keywords:** Trail Making Test, user experience, cognitive profile, information technology, task performance, cognitive assessment, human factors, cognitive function, CAPTCHA

## Abstract

**Background:**

Cognitive functional ability affects the accessibility of IT and is thus something that should be controlled for in user experience (UX) research. However, many cognitive function assessment batteries are long and complex, making them impractical for use in conventional experimental time frames. Therefore, there is a need for a short and reliable cognitive assessment that has discriminant validity for cognitive functions needed for general IT tasks. One potential candidate is the Trail Making Test (TMT).

**Objective:**

This study investigated the usefulness of a digital TMT as a cognitive profiling tool in IT-related UX research by assessing its predictive validity on general IT task performance and exploring its discriminant validity according to discrete cognitive functions required to perform the IT task.

**Methods:**

A digital TMT (parts A and B) named Axon was administered to 27 healthy participants, followed by administration of 5 IT tasks in the form of CAPTCHAs (Completely Automated Public Turing tests to Tell Computers and Humans Apart). The discrete cognitive functions required to perform each CAPTCHA were rated by trained evaluators. To further explain and cross-validate our results, the original TMT and 2 psychological assessments of visuomotor and short-term memory function were administered.

**Results:**

Axon A and B were administrable in less than 5 minutes, and overall performance was significantly predictive of general IT task performance (*F*_5,19_=6.352; *P*=.001; Λ=0.374). This result was driven by performance on Axon B (*F*_5,19_=3.382; *P*=.02; Λ=0.529), particularly for IT tasks involving the combination of executive processing with visual object and pattern recognition. Furthermore, Axon was cross-validated with the original TMT (*P*_corr_=.001 and *P*_corr_=.017 for A and B, respectively) and visuomotor and short-term memory tasks.

**Conclusions:**

The results demonstrate that variance in IT task performance among an age-homogenous neurotypical population can be related to intersubject variance in cognitive function as assessed by Axon. Although Axon’s predictive validity seemed stronger for tasks involving the combination of executive function with visual object and pattern recognition, these cognitive functions are arguably relevant to the majority of IT interfaces. Considering its short administration time and remote implementability, the Axon digital TMT demonstrates the potential to be a useful cognitive profiling tool for IT-based UX research.

## Introduction

Cognitive functional ability is a fundamental factor widely recognized to influence IT usability [1-3]. The classical approach to control for cognitive functional ability is to target participants according to general demographics based on age, education, or other factors [4,5]. However, this approach intrinsically precludes the ability to control for or assess how cognitive functional ability impacts IT usability in individual users, thereby limiting the extent which insight can be gained within a demographic or for an individual. Moreover, this approach is incongruent with the rapid advancement of IT toward products that adapt to individual user characteristics, thus necessitating a more granular understanding of individual cognitive abilities [6-8].

To obtain a granular characterization of individual cognitive function, hitherto, research has typically used cognitive assessment batteries [9-11]. Dumont et al [12] used the National Institutes of Health Toolbox, which is a battery of cognitive tests that can be completed in 40 minutes [13] to develop a cognitive analysis grid to be able to draw statistical parallels between the cognitive demands of an information systems interface and the performance of a user. Other batteries of tests were also used, such as the Kit of Factor-Referenced Cognitive Tests [10], which was used by Wagner et al [1] to study the impact of age on website usability and by Allen [14] in his research to study the combination of users’ cognitive abilities and specific information system functionalities that can be implemented to create system usability. This battery is typically administered in 144 minutes [15]. Another approach for assessing individual cognitive ability is to use clinically administered tests such as the Montreal Cognitive Assessment (MoCA) or the Mini-Mental State Examination (MMSE). Although typically used in medical settings to evaluate cognitive impairment in patients with neurological disorders [9,11], MoCA and MMSE have been reportedly used to measure the cognitive abilities of participants in human-computer interaction experiments [3,16-18]. However, while detailed and accurate, these cognitive assessment batteries are too lengthy to practically administer during typical user experience (UX) testing time frames [19,20]. Furthermore, while clinically administered tests such as MoCA and MMSE are comparatively shorter than other assessment batteries, they require a trained administrator to administer and score the test [3]. This level of expertise may not always be available, particularly in UX research settings where mostly nonclinically trained research personnel are conducting the experiments.

Correspondingly, there have been calls from across health, UX, and IT domains for a more practical yet accurate means of assessing cognitive function [12,21,22]. One solution would be to identify a short test with reduced scope but which nevertheless targets cognitive functions important for using IT. Based on research conducted to understand the impact of cognitive functions on the use of technology by older people [23,24], and on existing models of cognitive architecture in human-computer interaction [25], we identified 5 key cognitive functions important for IT use: visual perception, motor function, executive function, inhibitory control, and working memory. Visual perception is important for finding relevant information cues on a web page [23]. Motor functions are involved in tasks such as data entry using the keyboard, navigation using the mouse, or other tool to perform a digital task [26]. Executive functions come into play in order to make decisions and prioritize action [23]. Inhibitory control, also called “response inhibition” [27], is the functional ability to inhibit or override motor commands or other executive processing, such as when an external stimulus interferes with goal-driven behavior as in a task-switching situation [28,29]. Finally, short-term or working memory capacity may be important in IT task performance, for example, for remembering options or system output at a later stage [23].

One potential preexisting cognitive assessment candidate that targets these cognitive functions related to IT use is the Trail Making Test (TMT). First developed for the Army Individual Test Battery [30], the TMT is one of the most widely used instruments in neuropsychological assessment as an indicator of cognitive processing speed and executive functioning [31-35]. Many studies have been conducted to determine which cognitive abilities are engaged during the completion of this 2-part test (TMT-A and TMT-B). After a comprehensive review of the literature on the topic, Sánchez-Cubillo et al [36] explored the contributions of certain cognitive functions and found that part A of the TMT (TMT-A) mainly requires visual-perceptual abilities, and that part B (TMT-B) reflects primarily working memory, executive function, and task-switching ability. Finally, although its contribution in the TMT has been questioned by the study of Sánchez-Cubillo et al [36], it is interesting to note that psychomotor ability has been mentioned numerous times as one of the abilities required for both parts of the TMT (Groff and Hubble [37] in both parts, Schear and Sato [38], Gaudino et al [39], and Crowe [40] in part B). The primary objective of this study was to test the validity of using the TMT as a cognitive profiling tool to predict or explain the variance in IT task performance. With an interest in a practical tool for cognitive profile assessments in UX testing of digital artifacts, we chose to use a digital version of the TMT. To further support and explain our results, we additionally cross-validated the digital TMT with the original TMT, a visual search task assessing visuomotor processing [41,42], and a hidden path learning task assessing visuomotor-processing speed, spatial working memory, and error-monitoring ability [43]. We had two hypotheses: (1) TMT times would be predictive of general IT task performance and (2) that the predictive power of the TMT would be stronger for tasks requiring the use of cognitive functions that are congruent with those assessed by the TMT.

## Methods

### Sample

To test our hypothesis, we conducted a laboratory experiment with 27 healthy participants (12 men and 15 women), between 18 and 36 (mean 24, SD 4.22) years of age, who were mostly university students (n=22, 85%).

### Ethical Considerations

Written informed consent was obtained from all subjects via a signed form at the beginning of the experiment. This project was approved by our institution’s research ethics committee (#2021-4108). A monetary compensation of CAD $25 (US $18.35) was provided to each subject upon completion of the experiment. Data from 1 subject were lost due to technical issues, thus leaving data from 26 participants available for analysis. All data were anonymized prior to analysis and stored in encrypted servers only accessible by authorized researchers.

### IT Tasks

**Figure 1:**
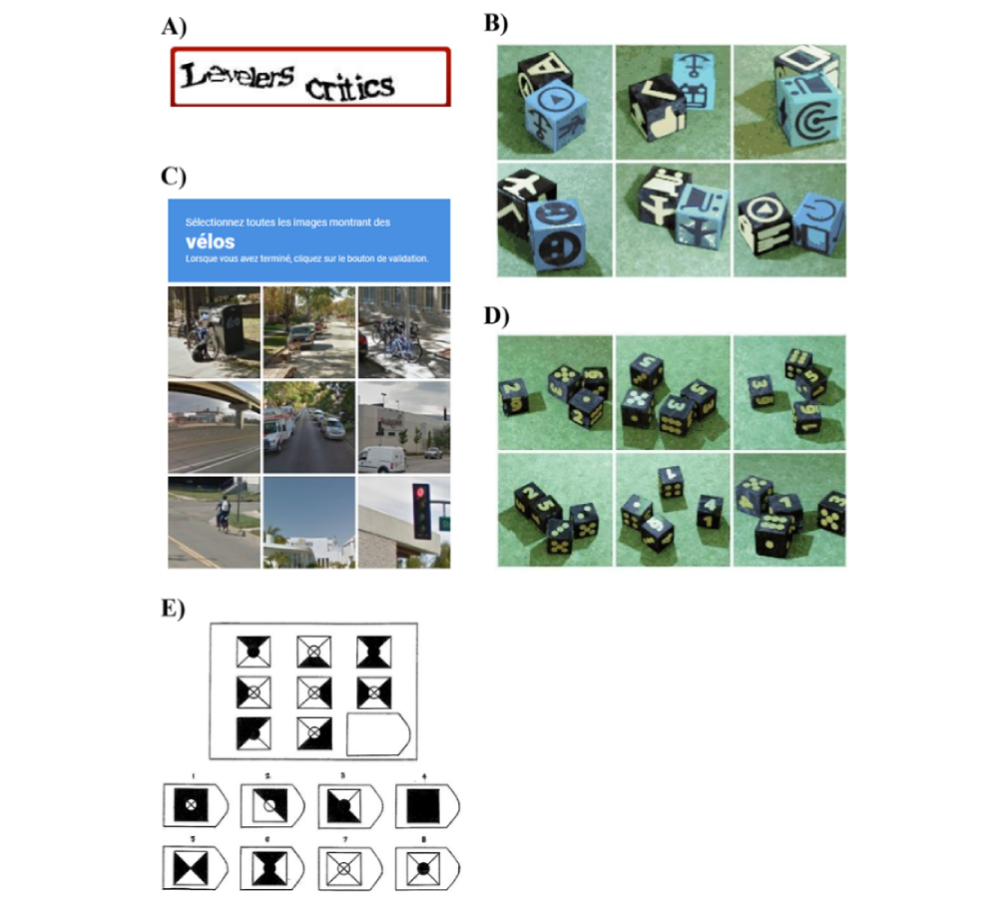
The 5 information technology tasks. (A) Text-based Completely Automated Public Turing tests to tell Computers and Humans Apart (CAPTCHA): subjects had to type the 2 words in an input field below the text image. (B) Pictogram recognition CAPTCHA: subjects had to recognize and click on the image showing the 2 dice with the same pictogram on the top face. (C) Google reCAPTCHA: subjects had to recognize and click on the images showing the bicycles. (D) Number recognition CAPTCHA: subjects had to recognize and click on the image showing dice summing to 14 on the top faces (numerals and dots combined). (E) Raven’s Progressive Matrix: subjects had to click from among the 8 proposed images the one which most appropriately fit in the missing corner of the basic matrix.

Two types of general IT tasks were used in the experiment. One type of IT task was based on CAPTCHA (Completely Automated Public Turing Tests to Tell Computers and Humans Apart). This type of Turing test is widely used in IT to ensure the cybersecurity of many internet services, as they prevent a number of attacks from automated programs (often referred to as bots), by distinguishing legitimate users from computer bots while requiring minimal effort by the human user [44]. Four CAPTCHAs were based on typical existent CAPTCHAs and included Google reCAPTCHA (Google), pictogram recognition (PicRec), numerical recognition (NumRec), and text recognition (Text). A Fifth task was taken from Raven’s Progressive Matrices (RPM) and presented in a CAPTCHA format. RPM are a collection of widely used standardized intelligence tests consisting of analogy problems in which a matrix of geometric figures is presented with 1 entry missing, and the correct missing entry must be selected from a set of answer choices [45]. A 3×3 RPM was selected as it was considered that it offered the best trade-off between cognitive effort and the time required to complete it. The final 5 IT tasks, shown in Figure 1, were embedded on a Qualtrics questionnaire. For this study, we targeted IT task completion time, measured as the time from the display of each task to when subjects responded and pressed the “next” button, based on 30 fps screen recordings.

The other type of IT task was a website design evaluation to assess perceived usability using Aladwani and Palvia’s [46] user-perceived web quality measurement scale. Screenshots of the home pages of the following 5 websites were used: Vignerons d’Exception [47], Renaud-Bray [48], LesPAC [49], [50], and [51]. One website was presented subsequent to each CAPTCHA. Participants were told that the website evaluation was the primary task of the experiment and that the CAPTCHAs were present as a security measure to access our database housing the website screenshots. However, the website evaluations were actually dummy tasks, and participant responses were not analyzed. The IT tasks really targeted and analyzed in this study were the CAPTCHAs.

### Cognitive Function Characterization of CAPTCHAs

The principal reason CAPTCHAs were chosen as our general IT tasks is because they are ubiquitous in IT and because they are often distinguishable from one another according to task-specific demands such as math, 3D orientation, text recognition, and visual search, suggesting that different underlying cognitive processing required them. However, there is a paucity of studies regarding the examination of the specific cognitive functions of CAPTCHAs. Therefore, we formed a panel of 11 trained, nonexpert evaluators to rank the selected CAPTCHAs on a 5-point agreement scale according to the 5 cognitive functions mentioned in the Introduction section, which have been deemed relevant to IT tasks and the TMT: visuospatial perception, motor function, executive function, inhibitory control, and working memory. The evaluation scores permitted each CAPTCHA to be assigned a rank according to the extent the cognitive functions required to perform it overlapped with those of the TMT. In order of highest to lowest alignment, the rankings were as follows: (1) RPM, (2) NumRec, (3) PicRec, (4) Google, and (5) Text, as shown in Table 1. For details of how this evaluation was conducted and how the process was validated, see Multimedia Appendix 1.

**Table 1:** Convergence ranks of IT tasks with the TMT^a^.

IT task	RPM^b^ (E)	NumRec^c^ (D)	PicRec^d^ (B)	Google (C)	Text (A)
Executive function, mean (SD)	5.00 (0.00)	4.91 (0.30)	4.45 (1.04)	4.45 (1.04)	3.82 (1.4)
Visual object recognition, mean (SD)	4.09 (0.70)	4.27 (0.65)	4.64 (0.67)	4.82 (0.60)	4.18 (1.17)
Visual pattern recognition, mean (SD)	4.91 (0.30)	4.45 (0.93)	4.64 (0.67)	3.82 (0.98)	4.64 (0.67)
Working memory, mean (SD)	4.18 (0.60)	3.91 (1.38)	2.91 (1.51)	2.45 (1.21)	2.73 (1.27)
Evaluation score for reliable convergent dimensions, mean (SD)	4.55 (0.48)	4.39 (0.42)	4.16 (0.84)	3.89 (1.04)	3.84 (0.81)
Convergence rank with TMT following the evaluation^e^	1	2	3	4	5

^a^TMT: Trail Making Test.

^b^RPM: Raven's Progressive Matrices.

^c^NumRec: numerical recognition.

^d^PicRec: pictogram recognition.

^e^Based on the average evaluation scores of IT tasks on the reliable cognitive dimensions considered convergent with the TMT. A, B, C, D, and E refer to the labels of the IT tasks presented in Figure 1.

### Digital TMT

**Figure 2:**
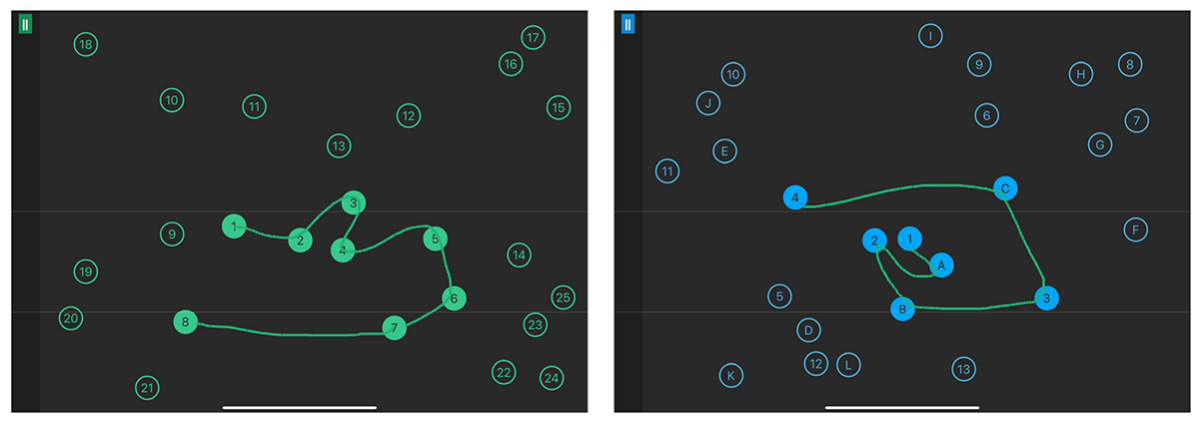
Screenshots of Axon A and Axon B. Subjects had to draw to connect the circles in ascending order (from 1 to 25 for part A and from 1 to 13 and A to L for part B, alternating numbers and letters) on a single line, without crossing paths or lifting their finger from the screen. In case of errors in drawing, the app automatically guided subjects back to the last correct circle from which they continued the test.

Because we are interested in cognitive assessment for UX testing of IT and because it was convenient to present all the tasks on the same device, we chose to use a digital version of the TMT called “Axon” (Language Research Development Group). This version emulates the original TMT as an iPad app, allowing the user to draw the trail on the touch screen with 1 finger. The 2 parts (A and B) of the TMT were completed, each with 25 circles to connect. Axon TMT was designed with a canvas generation algorithm, meaning that the test canvas for each subject for each TMT A and B was different. As shown in Figure 2, both tests were presented in full screen on the iPad with 25 circles of 1-cm diameter placed randomly on the digital canvas in a homogeneous way. The rules of Axon were identical to those of the original TMT, as outlined by Bowie and Harvey [52]. Participants had to connect the circles in ascending order: from 1 to 25 for part A and from 1 to 13 for part B, alternating numbers and letters in ascending order (ie, 1, A, 2, B, 3, C, etc). Errors such as lifting the finger off the screen, crossing trails, or connecting a wrong circle resulted in the line for the latest segment to be automatically erased and subjects had to return to the last successfully reached circle in order to continue. The measures chosen for this study were the completion time for each of the 2 parts of the test, from the moment the layout was displayed until the last circle was reached. These measures were exported from the app after the completion of the study and used in our statistical analyses.

### Cross-Validation of the Digital TMT

#### Overview

To better support and explain our results, we cross-validated Axon with the original TMT and a working memory and a visual search task.

#### Original TMT

The original TMT was administered as outlined by Bowie and Harvey [52] at the end of the study. The practice step was skipped in the interest of time and with the knowledge that the subject had already performed the digital TMT earlier in the study.

#### Hidden Path Learning Task

**Figure 3:**
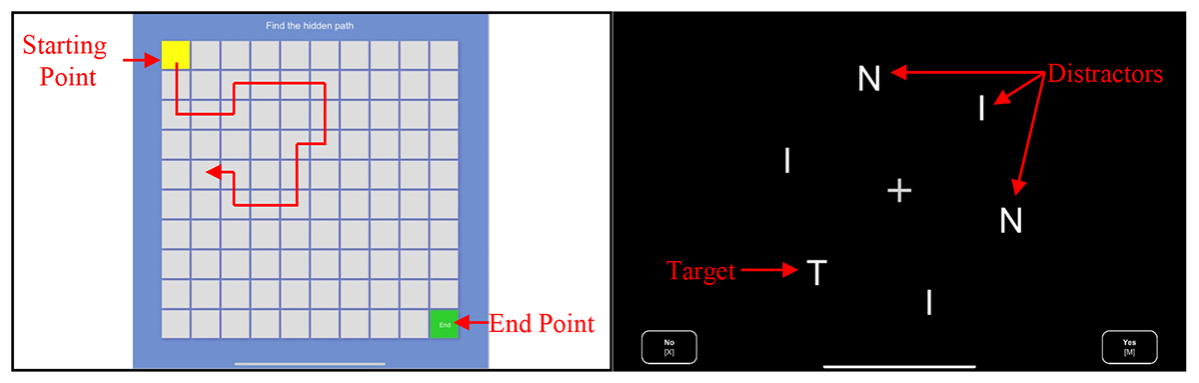
Cross-validation tasks. In the hidden path learning 10×10 matrix (left), subjects had to go from the yellow starting point to the green end point 1 tile at a time. In the visual search task (right), there were 6 items, with 1 target and 5 distractors. In the I+N sequence (shown), participants had to touch “Yes” at the bottom-right if they saw the target, “No” at the bottom-left otherwise.

To cross-validate Axon’s ability to measure working memory and spatial ability, we administered a hidden maze learning task, based on the Groton Maze Learning Test developed by Pietrzak et al [43]. Our task was called the “hidden path learning” task and was based on a 10 × 10 grid. Five trials were administered on the iPad via the Cognition Lab platform (BeriSoft, Inc), following similar guidelines as the Groton Maze Learning Test [43]. The hidden path learning task is particularly targeted at working memory, as the user has to call on it to navigate between tiles and remember any errors they may have made before [53,54]. Correspondingly, working memory ability is associated with the extent to which completion time decreases over trials, revealing a learning curve. Thus, the metrics used for these analyses were the difference between the completion times of each consecutive trial on the task. A depiction of the hidden path learning task is shown in Figure 3 (left). Measures were automatically collected on the Cognition Lab server.

#### Visual Search Task

To cross-validate Axon’s ability to measure visuomotor function, we administered a visual search task on the Cognition Lab platform (BeriSoft, Inc). This task was based on the work by Treisman and Gelade [42] and involved finding a target among distractors. Participants had to touch the right side of the screen when they saw the target, the left side otherwise, therefore involving visual and psychomotor response ability. Three stimuli configurations were used, with 3 distractor sets. Configurations were displayed with 24 trials for each stimulus, leading to a total of 72 trials. For each trial, 3, 6, or 9 symbols were displayed (letters or shapes), with even and randomized distribution among each stimulus sequence. A depiction of this task is shown in Figure 3 (right). Again, measures were automatically collected on the Cognition Lab server. Reaction times were used for the present analyses.

### Procedures

**Figure 4:**
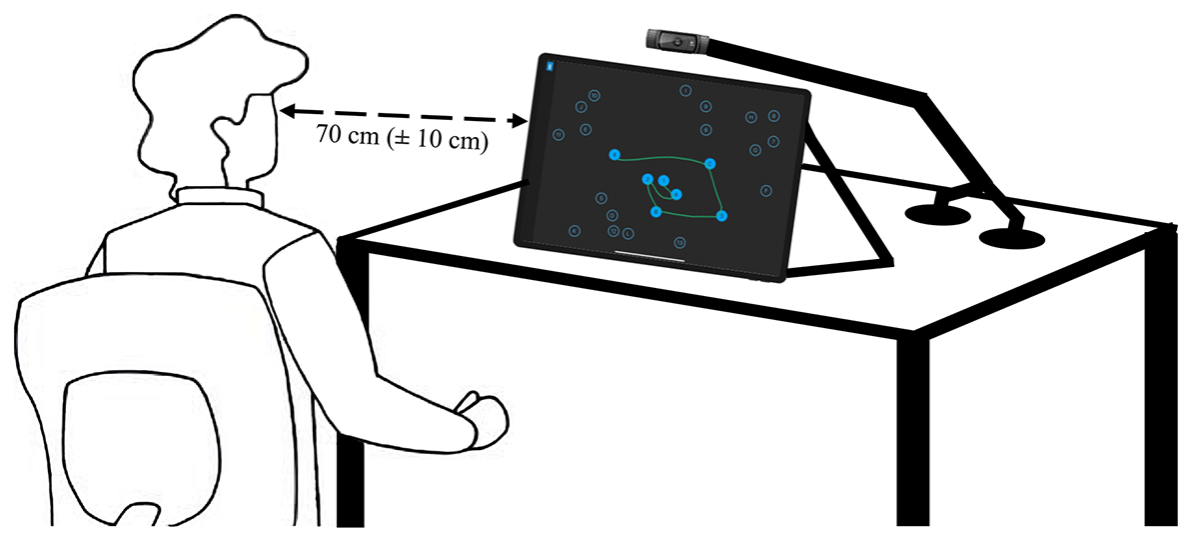
Experimental setup diagram. The subject was seated at a chair in front of a desk where the iPad Air 4 was placed. A Logitech C920 camera was independently fixed to the desk via a camera stand and duct tape.

Upon arrival and after signing the informed consent form, subjects were asked to sit on a chair facing the iPad Air (fourth generation) running on iPadOS 15.3 (Apple Inc) placed on a desk and were asked to adjust the chair’s height so that they were comfortable using the iPad, and they were within the camera recording frame. The experimental setup is presented in Figure 4. They were asked to move the chair closer or further away to maintain an approximate distance of 70 (±10) cm between their eyes and the iPad screen to give enough space for hand movement during the tasks. The camera was fixed independently from the iPad to avoid unwanted movements on the video when the participant presses the screen while doing the tasks. After a presentation of the study and the tools used, the participants were asked to complete the 2 parts of the TMT (A and then B) on the Axon app. Task instructions were given in a protocol format to ensure that all participants received the same instructions and that the data would be comparable. Participants were verbally and visually guided through the rules of the TMT using a tutorial embedded in the app.

After completing parts A and B on the Axon app, participants were administered the hidden path learning and the visual search tasks. Then, participants commenced the IT task portion of the experiment. As previously mentioned, participants were told that the primary objective was to evaluate 5 interfaces of more or less popular websites, each interface being on a secure server accessible only after the completion of a CAPTCHA. Thus, subjects completed a CAPTCHA, observed a web interface for a few minutes, and then completed the user-perceived web quality measurement scale [46]. This sequence was repeated 5 times, with the tasks presented in random order, each preceded by a distinct CAPTCHA. At the end of the study, for ethical reasons, subjects were told orally that they were in fact being evaluated on their performance on the CAPTCHAs.

### Statistical Analyses

To test the ability of the Axon TMT to predict performance on the 5 CAPTCHA IT tasks, a repeated-measures multivariate analysis of covariance (RM MANCOVA) was performed with Axon A completion time and Axon B completion time as independent predictors and the completion time for each of the 5 IT tasks as the dependent covariates.

To further interpret our results, we tested the relationship between Axon TMT completion times and visuomotor function by performing an RM MANCOVA with Axon A and Axon B times as independent predictors and the mean reaction time of each of the 3 visual search tasks (the shape of an arrow as a target among the triangle shapes as distractors, the letter T as a target among the letters I and N as distractors, and the letter T as a target among the letters I and Z as distractors) as the dependent covariates. In addition, we tested the relationship between Axon TMT completion times and working memory function by performing an RM MANCOVA with Axon A and Axon B times as independent predictors and the difference between the completion time of each consecutive trial on the hidden path learning task as the dependent covariates. Finally, we cross-validated the relationship between the Axon TMT and the original TMT using 2 Pearson correlation tests, 1 each for tests A and B.

For all RM MANCOVAs performed in the analysis, omnibus results and multivariate results for each independent predictor are reported. In the case of significant multivariate results, simple main effects based on parameter estimates are reported for dependent covariates, which were significantly predicted by Axon.

All statistical analyses were conducted using the IBM SPSS Statistics software (version 28.0.1.1; IBM Corp) with a threshold for statistical significance set at *P*≤.05, using the Bonferroni correction to adjust for multiple comparisons.

## Results

### Axon TMT Cross-Validation

#### Axon Versus Original TMT

The mean scores of Axon A and B were 48.04 (SD 25.80) and 56.88 (SD 25.53) seconds, respectively. The mean scores on the original TMT A and B were 29.22 (SD 12.26) and 51.62 (SD 19.07) seconds, respectively. Pearson correlation tests revealed that Axon is highly correlated with TMT results, with a significant positive correlation between Axon A and TMT A (*r*=0.688; *P*_corr_=.001) and a significant positive correlation between Axon B and TMT B (*r*=0.505; *P*_corr_=.017).

#### Axon TMT Versus Hidden Path Learning

The difference in consecutive trial times was (2–1) –29.87 (17.70), (3–2) –5.48 (6.01) seconds, (43) –4.30 (4.80) seconds, and (5–4) –1.50 (4.25) seconds. The omnibus test of the RM MANCOVA revealed that Axon A and Axon B combined are significant to explain the variance in the decrease in completion times across consecutive trials (*F*_4,20_=4.119; *P*=.01; Λ=0.548). However, multivariate results revealed that the decrease in completion times across trials was not predicted by Axon A (*F*_4,20_=1.923; *P*=.15; Λ=0.722) or Axon B (*F*_4,20_=1.106; *P*=.38; Λ=0.819) alone. Thus, a predictive relationship appears to exist between Axon and working memory in the hidden path learning task as a function of Axon A and B combined.

#### Axon TMT Versus Visual Search

Reaction times for the T among letters I and N, T among letters I and Z, and arrow among triangles were 0.80 (0.14) milliseconds, 0.78 (0.15) milliseconds, and 0.68 (0.14) milliseconds, respectively. The omnibus test of the RM MANCOVA revealed that Axon A and Axon B combined significantly explained the variance in visuomotor function assessed with reaction time to the 3 stimuli in the visual search task (*F*_3,21_=3.125; *P*=.048; Λ=0.691). Multivariate results revealed that this result was driven mainly by Axon A (*F*_3,21_=3.220; *P*=.043; Λ=0.685) rather than Axon B (*F*_3,21_=0.502; *P*=.69; Λ=0.933). Parameter estimates revealed that Axon A was marginally significantly predictive of reaction times to the letter T among letters I and N stimulus (β=3.573; t_21_=2.767; *P*_corr_=.055) and significant for letter T among letters I and Z (β=4.353; t_21_=3.156; *P*_corr_=.02) and arrow among triangles (β=3.725; t_21_=3.158; *P*_corr_=.02) stimuli.

### Axon TMT Predicts Overall IT Performance

**Figure 5:**
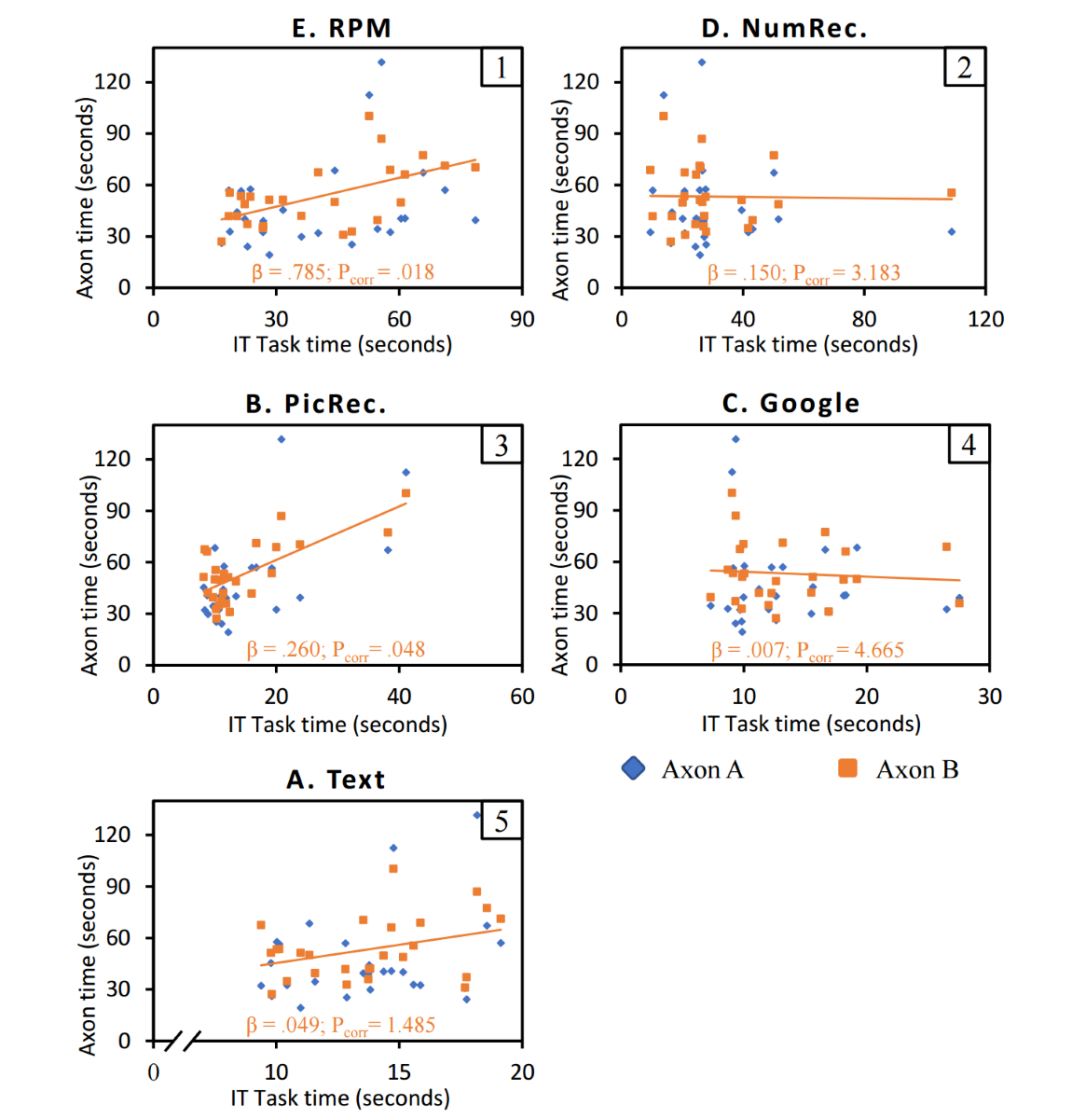
Distribution of Axon A and B completion times in relation with the completion times of the 5 IT tasks (N=26). Axon B trendlines and parameter estimates (β and P) show the relationship between Axon B IT task performance. Number in upper right corner of plot area is hypothesized convergence rank (Table 1). IT: information technology; NumRec: numerical recognition; PicRec: pictogram recognition; RPM: Raven’s Progressive Matrices. Letters A through E refer to the labels used for each task in Figure 1.

The primary hypothesis assumed that there was a positive predictive relationship between TMT performance and IT task performance. The omnibus test of the RM MANCOVA revealed that Axon A and Axon B combined significantly explain the variance in IT tasks performance (*F*_5,19_=6.352; *P*=.001; Λ=0.374), thereby supporting the primary hypothesis. Multivariate results revealed that this effect was driven by performance on Axon B (*F*_5,19_=3.382; *P*=.03; Λ=0.53). Figure 5 shows the distribution of Axon completion times in relation to IT task completion times.

### Axon TMT Better Predicts Performance on Convergent IT Tasks

The second hypothesis assumed that the predictive relationship between TMT performance and IT task performance would be stronger if the cognitive abilities involved in the performance were congruent. To test our hypothesis, we analyzed the parameter estimates for the multivariate results of Axon B. These revealed that Axon B was significantly predictive of IT task C (RPM task; β=.785; t_19_=3.240; *P*_corr_=.018) and IT task B (PicRec task; β=.260; t_19_=2.824; *P*_corr_=.048). However, IT task D (number recognition task), which was rated the second most congruent task with Axon, was not significantly predicted by Axon B (β=.150; t_19_=0.479; *P*_corr_=3.183). Our secondary hypothesis is therefore partially supported. These results are shown in Figure 5, where the effects of individual factors of Axon B on performance on IT tasks are represented (β and *P* values).

## Discussion

### Principal Findings

Cognitive functional ability may well affect task performance in UX and other research experimentation, leading to variance in performance measures among the target population and confounding the effects of experimental factors. Although detailed cognitive assessment batteries exist and can be used to control intersubject differences in cognitive abilities [12], they are not time efficient and thus impractical to implement within typical experimental time frames. Here, this study tested the validity of using the Axon TMT, which takes only a few minutes to administer, to predict or explain the variance in IT task performance in an age-homogenous subject population.

The mean age of the subject population of this sample was 24 (SD 4.22) years. This is typical of many research studies, UX related or otherwise, relying on student recruitment through the parent institution [55-57]. Despite the relatively low SD of age, the SD in Axon TMT scores was broad, at 25.80 (mean 48.04) and 25.53 (mean 56.9) seconds, respectively, for Axon A and B, suggesting a large distribution of cognitive functional abilities among this age-homogeneous neurotypical population. Notably, the means and SDs for the Axon TMT, particularly for Axon A, were higher than what is typically reported in the literature for neurotypical subjects in this age bracket [58-60]. This may be due to the fact that, unlike in the implementation of the paper-based TMT, subjects did not practice a mini version of the test before performing Axon A or B. Thus, some portion of the time taken to complete the test must be attributable to familiarization with task demands. This would also explain why the mean scores for Axon B, whose task demands are similar to Axon A in many respects, are closer to typically reported TMT B means. Nevertheless, for the purposes of this study, it is not absolute Axon TMT scores that are important. Rather, it is the relative distribution of the variance in Axon scores and their correlation to other metrics that is essential. To that end, both Axon A and B significantly correlated to their respective paper-based TMT counterparts showed a combined predictive validity toward working memory via the hidden path learning task. Furthermore, it was Axon A, not B, which was the predominant driver of the significant correlation with visual search performance. This is logical, as the visual search task does not involve working memory–related processing [42,61]. Instead, it requires an emphasis on target identification, cognitive control, and motor output, precisely the dominant cognitive functions involved in TMT A [36,39,40]. Thus, far from being problematic, implementing Axon A and B without a preliminary minitest for practice was time-efficient and yielded a reliable distribution of scores, which could be cross-correlated with expected cognitive functions.

This cross-validation lends credibility to our observation that Axon A and B combined were significantly predictive of IT task performance, supporting our primary hypothesis. Interestingly, for the IT tasks chosen, it was Axon B that appeared as the stronger driver of predictive validity, suggesting that it may be more powerful in capturing the executive decision-making involved in an ecologically valid IT task. Moreover, simple main effects tests revealed that Axon B significantly correlated with 2 out of the top 3 tasks ranked as requiring congruent cognitive functions as the TMT, thereby partially supporting our secondary hypothesis. Contrary to our expectations, the NumRec task, which had the second-highest congruence rank, was not significantly correlated with Axon B. We speculate that the confound here relates to the underlying mathematical operations involved in solving that CAPTCHA. Although raters classified this as executive decision-making, it certainly can be said that neither TMT A nor TMT B requires arithmetic. Therefore, there must be cognitive processes involved that are simply not recruited during the performance of the TMT, which our ranking system was not granularized enough to capture, hence explaining the lack of correlation between the NumRec task and Axon B. Meanwhile, Axon B was most strongly correlated with the RPM and PicRec task, suggesting that it is well suited for tasks involving visual pattern and object recognition in combination with higher-order executive processing to orient this visual information. These kinds of processing are arguably crucial for interface navigation, virtual reality, gaming, or using simulators, which are extremely common IT tasks investigated in UX research [62-64]. Thus, while Axon does appear to be better aligned with IT tasks involving convergent cognitive processing, such tasks may well comprise a major proportion of those studied in UX research.

Finally, there are a few points worth emphasizing. First, the complete administration of Axon took less than 5 minutes, far shorter than the strategy used by Dumont et al [12] or any other cognitive assessment that we are aware of. Second, considering Axon’s ability to differentiate from among an age-homogeneous neurotypical population, it would likely perform even better among populations where a larger variance in cognitive function would be expected, such as in older adults, children, stroke survivors, or other individuals with atypical cognitive function. This is important because understanding how to design appropriate and accessible IT for these populations has become a topic of increasing concern in UX research [65-67]. Moreover, Axon is suitable for remotely moderated experimentation, a popular strategy since the COVID-19 pandemic [68] and one that mitigates subject recruitment challenges for all population types. Finally, the current advancement in technology, particularly in the field of artificial intelligence, is trending toward a more personalized and user-centric approach, adapting technology to individual user characteristics such as preferences and interests [8,69,70]. Part of this personalization could be to tailor technology according to the cognitive abilities of users. Axon could potentially facilitate this advancement, serving as a quick and reliable metric to train the artificial intelligence technology adaptation algorithm.

### Limitations

There are some limitations that should be acknowledged with this study. First, because the Axon app is designed to produce TMT canvases according to an algorithm with every test instance, the Axon A and B canvas layouts were not constant across subjects. This means that some of the variance in Axon A and B times is intrinsically attributable to factors such as differences in the straight-line drawing path length of the test or the extent of visual interference between each drawing segment. On the other hand, the fact that Axon A and B were significantly cross-validated with the original TMT and the visual search and hidden path learning tasks in spite of canvas layout differences between participants suggests that the variance these differences cause is small and does not detract from the use of Axon as a cognitive profiling tool in UX testing. Second, this study tested the predictive validity of Axon on simple and discrete IT tasks. This was necessary as a proof of concept for our hypotheses. However, readers should use caution when generalizing the present results. Further research is needed to investigate the extent to which Axon retains predictive validity for more complex IT tasks in different contexts and across various user demographics, including neuroatypical and cognitively impaired users.

### Conclusions

This study tested the ability of the Axon digital TMT to predict performance on discrete IT tasks. The results indicate that variance in IT task performance among an age-homogenous neurotypical population can be related to intersubject variance in cognitive function as assessed by Axon. Although the findings suggest that Axon’s predictive validity may be strongest for IT tasks involving the combination of decision-making with visual object and pattern recognition, these types of cognitive processing would arguably be relevant to the majority of IT interfaces. Considering its short administration time and remote implementability, the Axon digital TMT has the potential to be a useful cognitive profiling tool for IT-based UX research.

## Appendix

Multimedia Appendix 1 Evaluation of CAPTCHAs–congruent cognitive functions in the information technology tasks.

## Abbreviations

CAPTCHA: Completely Automated Public Turing tests to tell Computers and Humans Apart
MMSE: Mini-Mental State Examination
MoCA: Montreal Cognitive Assessment
NumRec: numerical recognition
PicRec: pictogram recognition
RM MANCOVA: repeated-measures multivariate analysis of covariance
RPM: Raven’s Progressive Matrices
TMT: Trail Making Test
UX: user experience
