# Supplementary material for: Assessing the Relationship Between Digital Trail Making Test Performance and IT Task Performance: Empirical Study
Source: JMIR Hum Factors. 2024 Jun 14;11:e49992. doi: 10.2196/49992 (PMC11214028; doi:10.2196/49992)
Supplement: Multimedia Appendix 1 [file humanfactors_v11i1e49992_app1.docx]

**Multimedia Appendix 1.** Evaluation of CAPTCHAs–congruent cognitive functions in the information technology tasks.

An evaluation was conducted prior to the study to confirm our assumptions about the cognitive abilities involved in the performance on the 5 computer tasks we chose to present as CAPTCHAs. The evaluators were 11 graduate and postgraduate students from our user research lab (9 female and 2 male evaluators, mean 25 , SD 2.21 years). The goal of this evaluation was to assess the extent to which different cognitive functions were needed to perform the CAPTCHAs used as IT tasks in this study. Evaluators were trained by the authors, then performed the CAPTCHAs, and rated them on a 5-point Likert scale according to the following cognitive functions the introduction highlighted as relevant for general IT tasks and the TMT: visual perception, motor function, executive function, inhibitory control, and working memory. Visual perception was subdivided into 2 functions: visual object recognition and visual pattern recognition. Evaluators were provided detailed descriptions of each of the 7 cognitive functions and reviewed each of them with the authors. The IT tasks and their evaluation grids were presented randomly to limit the risk of bias. Assessments were recorded using the online survey tool Qualtrics.

An intraclass correlation analysis was conducted to investigate the reliability of the evaluators in each group. Intraclass correlation coefficients (ICC) for the means of each cognitive ability scores are presented in Table 1 below. After the intraclass correlation reliability analysis, only reliable cognitive abilities were considered for further analysis. Each CAPTCHA was assigned a TMT convergence rank according to its mean evaluation score across all reliably evaluated cognitive functions.

| **Table 1.** Convergence ranks of IT tasks with the TMT. | | | | | | |
| --- | --- | --- | --- | --- | --- | --- |
| **Evaluation category** | **RPM** | **NumRec** | **PicRec** | **Google** | **Text** | **Reliability (ICC; *P*)** |
| Executive function | 5 | 4.91 | 4.45 | 4.45 | 3.82 | .756; .001 |
| Visual object recognition | 4.09 | 4.27 | 4.64 | 4.82 | 4.18 | .557; .043 |
| Visual pattern recognition | 4.91 | 4.45 | 4.64 | 3.82 | 4.64 | .740; .002 |
| Working Memory | 4.18 | 3.91 | 2.91 | 2.45 | 2.73 | .911; <.001 |
| Motor function^a^ | 3.09 | 3.27 | 3.27 | 3.82 | 3.27 | .252; .254 |
| Inhibitory control^a^ | 3.36 | 3.18 | 2.64 | 2.64 | 3.09 | -.95; .501 |
| **Mean evaluation score for reliable convergent dimensions** | **4.55** | **4.39** | **4.16** | **3.89** | **3.84** |  |
| **Convergence rank with TMT following the evaluation^b^** | **1** | **2** | **3** | **4** | **5** |  |
| ^a^Not used for convergence ranking due to reliability. b: Based on the mean evaluation scores for reliable categories. ICC: intraclass coefficients. | | | | | | |
